# Cultural Adaptation of a Scalable World Health Organization E-Mental Health Program for Overseas Filipino Workers

**DOI:** 10.2196/11600

**Published:** 2019-03-29

**Authors:** Melissa R Garabiles, Melissa Harper Shehadeh, Brian J Hall

**Affiliations:** 1 Ateneo de Manila University Manila Philippines; 2 Global and Community Mental Health Research Group The University of Macau Macau China; 3 Institute of Global Health Faculty of Medicine University of Geneva Geneva Switzerland; 4 Department of Health, Behavior and Society Johns Hopkins Bloomberg School of Public Health Baltimore, MD United States

**Keywords:** cultural adaptation, migrant workers, e-mental health, overseas Filipino workers

## Abstract

**Background:**

Electronic mental (e-mental) health interventions can address mental health needs of different populations. Cultural adaptation of these interventions is crucial to establish a better fit with the cultural group and to achieve better treatment outcomes.

**Objective:**

This study aimed to describe the cultural adaptation of the World Health Organization’s e-mental health program Step-by-Step for overseas Filipino workers. We used a framework which posits that cultural adaptation should enhance (1) relevance, wherein the cultural group can relate with the content; (2) acceptability, where the cultural group will not find any element offensive; (3) comprehensibility, where the program is understandable; and (4) completeness, wherein the adapted version covers the same concepts and constructs as the original program. We aimed to have English and Filipino and male and female versions.

**Methods:**

Overall, 3 experienced Filipino psychologists provided their perspectives on the program and how it might be adapted for overseas Filipino workers. We then adapted the program and obtained feedback from 28 overseas Filipino workers from diverse industries through focus group discussions. We conducted 7 and 9 focus group discussions with male and female participants, respectively. Per discussion, cognitive interviewing was used to probe for relevance, acceptability, comprehensibility, and completeness of illustrations and text. Participant feedback guided iterative program adaptations, which were again shown to participants for validation and improvement.

**Results:**

Several issues were raised by participants about the generic version of Step-by-Step. There were elements deemed irrelevant, like unfitting characters, lack of Filipino values, and unsuitable problems and activities. There were unacceptable components that were stigmatizing, political, inappropriate to context or subgroups, and too feminine for male users. Some elements were incomprehensible, unclear, or complicated. To address these issues, we made key adaptations. To enhance relevance, we adapted the narrative to match the experiences of overseas Filipino workers, incorporated Filipino values, and illustrated familiar problems and activities. To increase acceptability, our main characters were changed to wise elders rather than health professionals (reducing mental health and help-seeking stigma), political or unacceptable content was removed, and the program was made suitable for overseas Filipino workers from different sectors. To increase comprehension, we used English and Filipino languages, simplified the text to ease interpretation of abstract terms, and ensured that text and illustrations matched. We also used Taglish (ie, merged English and Filipino) when participants deemed pure Filipino translations sounded odd or incomprehensible. Finally, we retained the core elements and concepts included in the original Step-by-Step program to maintain completeness.

**Conclusions:**

This study showed the utility of a 4-point framework that focuses on acceptance, relevance, comprehensibility, and completeness in cultural adaptation. Moreover, we achieved a culturally appropriate adapted version of the Step-by-Step program for overseas Filipino workers. We discuss lessons learned in the process to guide future cultural adaptation projects of e-mental health interventions.

## Introduction

### Background

The use of technology to deliver mental health interventions proliferated in recent years, with electronic mental (e-mental) health interventions providing accessibility to needed mental health interventions [1,2] for more people [3] and at greater frequency [4]. The use of e-mental health interventions is especially promising for vulnerable and marginalized populations such as migrants. They help reduce the stigma associated with help-seeking and minimize treatment barriers such as geographical distance and culture, religion, and language differences between users and providers [2,5].

E-mental health interventions that undergo cultural adaptation are more effective. Cultural adaptation is “the systematic modification of an evidence-based treatment (EBT) or intervention protocol to consider language, culture, and context in such a way that is compatible with the client’s cultural patterns, meanings, and values” [6]. Cultural adaptation is warranted when an intervention developed for one cultural group will be implemented within a different cultural group. When an intervention is adapted, a better fit between the program and the cultural group is expected, which in turn leads to better treatment outcomes [7].

Culturally adapted interventions are effective. A meta-analysis [8] of 78 studies revealed that culturally adapted face-to-face interventions performed better than the comparison conditions (another active intervention or no intervention), with an average effect size of *g*=.67. Furthermore, culturally adapted interventions are more effective than their original unadapted versions, with a medium effect size of *g*=.52. Another meta-analysis [9] of e-mental health interventions found that cultural adaptation resulted in a greater reduction in depression and anxiety symptoms. Essential elements for adaptation according to the Bernal and Saez-Santiago framework [10] include language, persons, metaphors, content, concepts, goals, methods, and context. Every additional element adapted resulted in a 14% increase in intervention efficacy.

It should be noted, however, that cultural adaptation does not equate with completely rewriting the program. In fact, a systematic review [11] on cultural adaptation of interventions for depressive disorders showed that all the studies included in the review preserved the original treatment’s framework and core principles that were deemed acceptable. The adaptations were made to establish cultural relevance, improve treatment acceptability, and remove barriers to care (eg, lack of trained professionals and limited literacy). Inclusion of barriers to care could be considered as implementation rather than a cultural aspect and suggests that in some studies what counts as a cultural adaptation may be poorly defined.

Cultural adaptation involves an integration of top-down and bottom-up approaches [12]. The original program (top-down) is modified based on feedback from the intervention target population (bottom-up), that is, the original program is adapted based on input from the cultural group to be responsive to the cultural group’s context and specific mental health concerns [8].

The World Health Organization is developing several evidence-based interventions—including transdiagnostic programs—designed to be scaled up to reach populations globally that lack access to needed mental health services [13]. We adapted one of these, the Step-by-Step program, an e-mental health program based on the principles of behavioral activation treatment for depression, along with additional strategies such as psychoeducation, stress management, and help-seeking [14]. Following the internet- and mobile-based intervention categorization [1], Step-by-Step is considered a minimally guided self-help program, wherein a nonspecialist *eHelper* may provide technical support and assistance in accomplishing program activities through phone calls or text messaging for up to 20 min a week.

In places with more established services, Step-by-Step may be suitable for use within a stepped-care structure, where users who show mild-to-moderate levels of symptoms could be referred to higher-intensity services should they require it on completion of the program. The program contains text and stories with corresponding illustrations and 2 main characters. The first character is an expert who helps the second character by sharing behavioral techniques to overcome problems and by explaining the concepts behind the problems and the techniques offered in the program. The second character narrates their experience of using the Step-by-Step techniques to overcome depression, reporting their previous encounters with typical problems and psychological symptoms of the target population and explaining how they employed the program to reduce these problems.

The original version already has 3 of the 4 desired features of effective e-mental health interventions, as it is based on the empirically tested theory of behavioral activation, it is structured, and it is interactive or experiential [15]. The last desired feature of being targeted for a specific group [15] must be addressed through cultural adaptation. To ensure high-quality adaptation of Step-by-Step, we followed the approach used by Manson [16] and van Ommeren et al [17]. Manson [16] theorized that the end product needs to be (1) acceptable, with nothing in the program offensive or potentially offensive to the cultural group; (2) relevant, in that the program’s content is related to the cultural group and does not contain unrelated phenomena; (3) comprehensive or understandable by the cultural group; and (4) complete, in that the program covers the same semantics, concepts, and theoretical constructs as the original version.

In this study, Step-by-Step was adapted for overseas Filipino workers (OFWs). There are 2.24 million OFWs around the world [18]. The majority (85%) work in Asian countries such as Saudi Arabia, United Arab Emirates, Qatar, Singapore, and China (ie, Macao and Hong Kong Special Administrative Regions, SARs). There are roughly the same number of male and female OFWs, but occupational differences exist between the groups. Male OFWs are mostly plant and machine operators and assemblers (24.7%) and craft and related trade workers (23.1%), whereas female OFWs are mostly employed as household service workers and cleaners and in other low-skilled occupations (56.2%). In Macao SAR of the People’s Republic of China, OFWs are the second largest migrant group at 29,426 [19]; roughly half of them are household service workers, more commonly referred to as domestic workers, followed by hotel and restaurant workers.

Previous studies focused on the risks and challenges that OFWs and other labor migrants experience. OFWs’ primary reason for working abroad is the desire to escape poverty or to achieve socioeconomic mobility, mainly for their family, rather than individual aspirations [20,21]. However, while abroad, and similar to other labor migrants, OFWs are at higher risk of experiencing mental health–related issues such as loneliness, stress, anxiety, depression, and serious mental illness [21,22]. However, resolving these challenges is problematic as labor migrants tend to have poor access to mental health services [23-25] and poor support systems [26]. E-mental health interventions are a way to address their mental health needs as more than 90% are smartphone users, and there is high potential uptake as 68% are likely to use a Web-based program when one is available [27].

### Objectives

This study aimed to culturally adapt the WHO Step-by-Step program for overseas Filipino labor migrants. We made English and Filipino and male and female versions. It was intended for use by OFWs engaged in different occupations.

## Methods

### Participants

**Figure 1:**
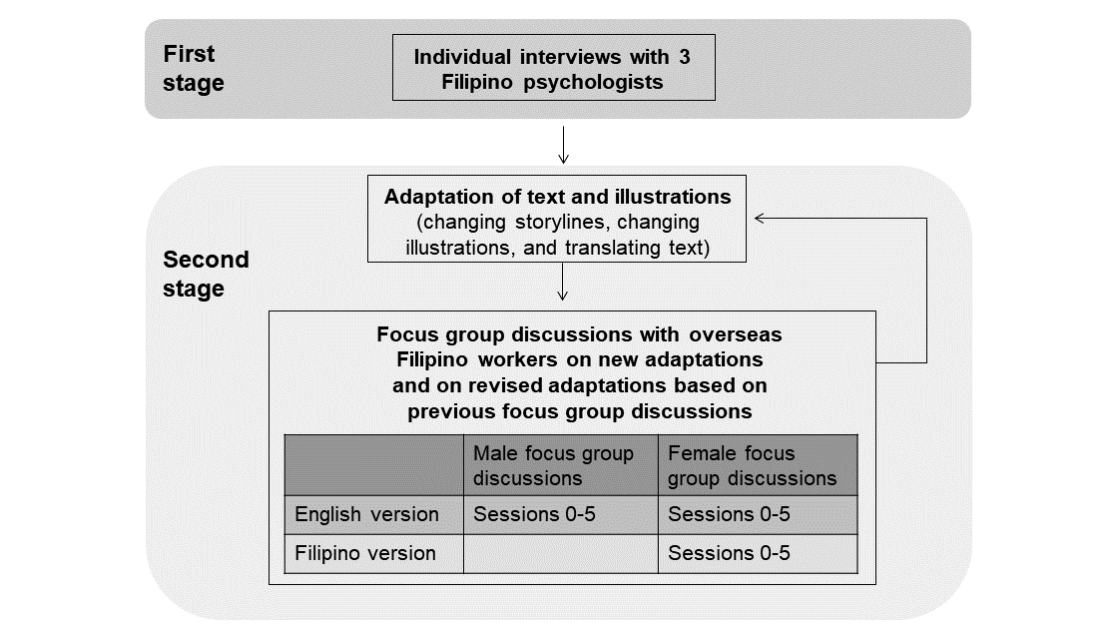
Flow of the adaptation process.

There were 31 participants, all of whom were selected through purposive sampling. We used a 2-stage approach (refer to Figure 1), first interviewing 3 Filipino psychologists with considerable experience and expertise in psychological practice and in working with OFWs, using Zoom video conferencing (each approximately 120 min long). The second stage consisted of focus group discussions (FGDs) with 28 OFWs in Macao. Of the FGD participants, 16 were women, aged 24 to 52 years (mean 36.31, SD 9.44), and employed as domestic workers, caregivers, and food and beverage workers. In addition, 9 of them were married, 6 were single, and 1 was widowed. The length of time working in Macao ranged widely, from 1 month to 12 years (mean 4.28 years, SD 3.46). Furthermore, 12 participants were men, aged 23 to 47 years (mean 30.58, SD 7.66), and employed in hotel and casino, food and beverage, and facilities management industries. Half of them were single and half were married. They had been working in Macao for an average of 1.63 years (SD 1.12), with a range of 6 months to 4 years. FGDs were conducted separately for male and female participants, with each FGD covering 1 to 3 sessions of the program. Due to scheduling difficulties, participants were not able to join all FGDs to discuss all the sessions of the program. Each participant joined at least 1 FGD and at most 6 FGDs. There were 7 male and 9 female FGDs in all, with 2 to 11 participants per FGD. The FGDs lasted between 2 and 3.5 hours and were conducted in private rooms in a local nongovernmental organization (NGO) or in a university. The FGDs were held on Saturday nights and Sundays, either after participants’ work or during their day off. The participants were remunerated MOP $100 or roughly US $12 per FGD.

### Materials

The intervention material was a generic version of the Step-by-Step program. The program starts with registration and introduction (session 0), followed by sessions 1 to 5. Each session is meant to be completed on the Web by the user once per week, with each session lasting between 30 and 40 min.

In advance of the interviews with expert Filipino psychologists, we provided a summary of the program’s content, the complete intervention content, and examples of possible illustrations with instructions to review all materials before the interview. For the FGDs with OFWs, we made Microsoft PowerPoint presentations that showed the entire initial adapted texts and illustrations (after Filipino psychologist interviews). Each slide consisted of about 3 to 5 sentences, most with simple illustrations. We started only with the intervention text for sessions 0 to 2, as the illustrations were not yet ready. Eventually, we showed both texts and illustrations to the participants side by side for the remaining 3 sessions.

We also utilized interview and FGD guides. The interview guide for the psychologist interviews consisted of questions about their opinions on whether or not the Step-by-Step program can address OFWs’ mental health needs, the type of OFWs that the program will be most or least suitable for (ie, hotel staff and domestic workers), appropriateness of the content of the program, considerations we had to make with regard to Filipino culture and OFW culture, and challenges they foresaw in using the program. We added questions on which specific groups we needed to consider for content tailoring (eg, younger or older OFWs), suggestions on the characters in the story, and opinions with regard to the characteristics of the eHelpers.

For the FGDs with OFWs, we started *cognitive interviews* (a technique whereby participants *think aloud* [28]) with a broad question on what participants thought about each of the PowerPoint slides. We then probed, using open-ended questions, if the content is relevant or relatable, understandable, or acceptable and the ways by which we can improve the text or illustrations. We also asked if the text and illustrations on each of the slides match, and if not, how we can change the text or illustrations to ensure they correspond with each other. When asking these questions, we told participants to think about all OFWs globally (eg, Would all or most OFWs understand this text?) and not just themselves or just OFWs in Macao to increase generalizability to OFWs across ages, marital statuses, occupations, and countries where they are employed.

### Procedure

FGDs were conducted in English to adapt an English-language version and in Filipino to adapt a Filipino-language version. We matched the sex of the facilitator with the sex of the participants (ie, male facilitator during male FGDs).

We first gave participants consent forms to read and sign, and then, we introduced the interview or group discussion and the Step-by-Step program. We then proceeded with the interviews or FGDs. The first FGDs concerned developing the illustrations of the main characters. After this, once illustrations had been developed, further groups were conducted based on the remainder of the stories and activities. The entire adaptation process was thoroughly documented. We used note-taking, wherein we wrote participants’ feedback for each slide. We also used audio recording to review the sessions if any of our notes were unclear. After an interview or FGD, we (MRG and BJH) discussed what transpired and reviewed our notes. We decided on pertinent changes that needed to be made on each slide. Throughout the process, we used the approach of Manson [16] and van Ommeren et al [17] in that we focused on adaptations that would better ensure relevance, acceptability, and comprehensibility to as many OFWs as possible, while retaining completeness of the Step-by-Step program. When participants’ opinions differed in terms of whether to make changes or not (ie, a sentence was understandable to some participants but others suggested to simplify or provide more explanation), we decided to make adaptations to ensure the content was appropriate to the broadest possible audience, and these modifications were then brought back to the community for any further comment and approval. When there were varied suggestions on how to adapt text and illustrations (ie, different suggestions on what words to use or how to change illustrations), we chose what the majority preferred and what was applicable to most OFWs (ie, applicable to the majority of male and female OFWs, young and old).

All changes in text and illustrations were documented using standard forms developed by the World Health Organization. We deemed making changes based on our notes sufficient because participant feedback on the slides was simple and straightforward (ie, participants mentioned which sentences were confusing, and they shared which part of the illustrations did not match the text), and participants provided us with concrete suggestions on what changes to make (ie, they suggested words to use and what to add or omit in the illustrations). Furthermore, each slide contained relatively short text and simple illustrations to begin with. The questions we asked them were also direct (ie, Can most OFWs understand the text? How might we improve the text to make it more understandable? Can you relate to the character? How might we make the character more relatable?).

Suggested changes to the illustrations were sent to a professional illustrator. He was the same illustrator who made the illustrations for the original Step-by-Step program and was familiar with the program and cultural adaptation process. Using the original illustrations as the starting point, we gave instructions on what changes to make (ie, character’s facial expression should be happier or omit certain hand gestures) and at times accompanied instructions with sample photos taken from the internet to guide the development of new illustrations.

**Figure 2:**
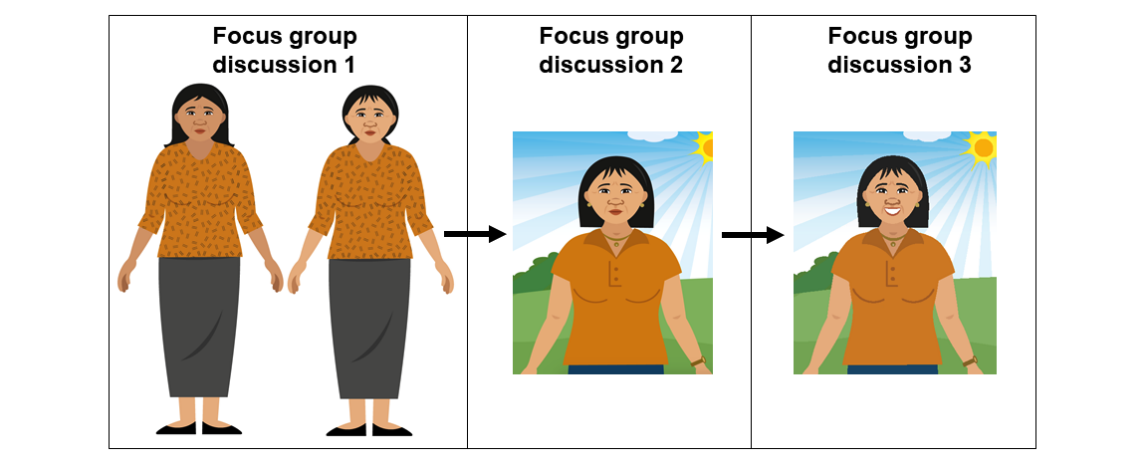
Character adaptations. In focus group discussion (FGD) 1, two sample illustrations of the main character were presented to participants for feedback with regard to appearance. Participants gave vivid descriptions on how they wanted her to look (ie, short hair with bangs, with jewelry, etc) In FGD 2, revised illustration based on FGD 1 feedback was shown to participants who gave partial approval. They liked how the character looked but not her personality. Participants suggested making her appear warm and happy. In FGD 3, revised illustration based on FGD 2 feedback was shown to participants, who then gave full approval.

Changes in illustrations and in stories were then shown to participants during subsequent FGDs. We reminded participants of their comments on the past illustrations and text and showed them the revisions made. We asked for their feedback on the changes and then their approval (ie, Is this what you meant? Anything else that is unrelatable, unacceptable, or hard to understand?). If the changes were still unsatisfactory, we asked for more input from them, made necessary revisions, and then showed them the newly revised slides in the next FGD. For example, it took 3 FGDs to finalize the appearance and get participants’ approval for one of the characters (refer to Figure 2).

After we conducted all the FGDs on the English and female versions and after revising the texts, we sent the texts to a professional translator. The translator was a Filipino fluent in both Filipino and English, with a BA in *Malikhaing Pagsulat* or Filipino creative writing. We gave her a background of the adaptation process and informed her of the desired tone of the story and personality of the characters as per the interview and focus group feedback. We then edited the translated text to capture the OFW experiences better (ie, words they often used in FGDs to describe their experiences). The edited translations were then shown to female FGD participants during subsequent cognitive interviewing. Their comments and suggestions to simplify the texts, especially words that were too difficult to understand or lines that were too long, were then used to make additional translation edits. We then showed the revised slides during the next FGD for feedback and approval.

### Reflexivity and Procedures for Verification

This study was conducted by a research group that aimed to improve the health of migrants globally and within the Macao SAR. As such, the implementing researchers (MRG and BJH) were familiar with the OFW context, through prior knowledge and interactions with the study population, and worked with organizations providing support to this community (eg, Philippine Government, churches, and NGOs) for years, including those based in the Macao SAR. MRG is a native Filipino who speaks the Filipino language fluently and has a similar cultural background as the participants. During data collection, MRG was an OFW herself but was only involved in the project as an investigator but not a participant. BJH is from the United States of America and emigrated to China over 5 years ago. However, neither researcher is employed in the same sector as the participants. We also had higher educational attainment, occupational prestige, and socioeconomic status. Although we are migrants ourselves and have a rich understanding of the OFW context, our experiences are different from the participants’ and other typical OFWs. With this acknowledgment, we attended to our own beliefs by assuring participants their feedback on the materials were crucial in improving the program and that they were the experts when it came to OFW experiences. We also probed participants’ answers to get more information and asked them to confirm our understanding of their input. Furthermore, we presented to them the interim changes we made on the stories and illustrations for validation. They verified many of the changes, but at times, they provided clarifications and more details for additional changes. We also presented the findings to a group of stakeholders, which included Philippine Government staff (Consul General, Labor Attaché), NGO workers, a Macao government official, Macao mental health professionals, and Filipino priests and nuns, for their feedback. They did not suggest changes in the adapted program and instead raised concerns regarding logistics and eHelpers.

## Results

### Acceptability

On the basis of key informant interview data and FGD data, there were 3 main issues with regard to the acceptability of the generic version of Step-by-Step. This includes stigma regarding mental health problems and mental health help-seeking in the Philippines. On the basis of interview data with experts, the use of a doctor pathologizes the users’ experiences and connotes there is something wrong with them. This would then serve as a barrier for the OFW population to use the program. This opinion is also backed by previous research [29]. Another issue was, from the participants’ viewpoint, that some of the original content was politically charged or socially unacceptable to all OFWs. For example, the initial illustrations included hand gestures that reminded participants of political parties. The text also mentioned drug use as a negative coping strategy, but participants shared that this was reminiscent of the popular but polarizing drug war campaign of the current administration in the Philippines. Another example was illustrations that depicted the characters doing household chores, which leaned too much toward domestic work. Although many OFWs are skilled workers, there are many professionals such as nurses, managers, and teachers as well, which makes doing household chores unsuitable for all OFWs. Other instances were coping strategies originally depicted as negative but are normative or even positive coping strategies in the OFW context. For example, an illustration and text were about a character staying in bed all day, but participants shared that for OFWs in physically demanding jobs, this is a good strategy to recover from stress at work. Another was *drinking alcohol* because it is culturally normative for Filipinos to drink, especially when they are with their friends. Finally, some male participants mentioned that some text and illustrations looked too feminine (ie, crying in bed). Due to strong traditional gender roles, it was important for them to maintain a masculine stance or at least not exude femininity, including emotional weakness.

To address these issues raised by participants, adaptations were then made to make Step-by-Step acceptable. These include reducing content that may increase the stigma associated with mental health and help-seeking, changing content that appeared political or potentially unacceptable to an OFW subgroup and depicted as negative when it is socially acceptable in the OFW’s context, and making considerations for the male version. These are explained below.

**Figure 3:**
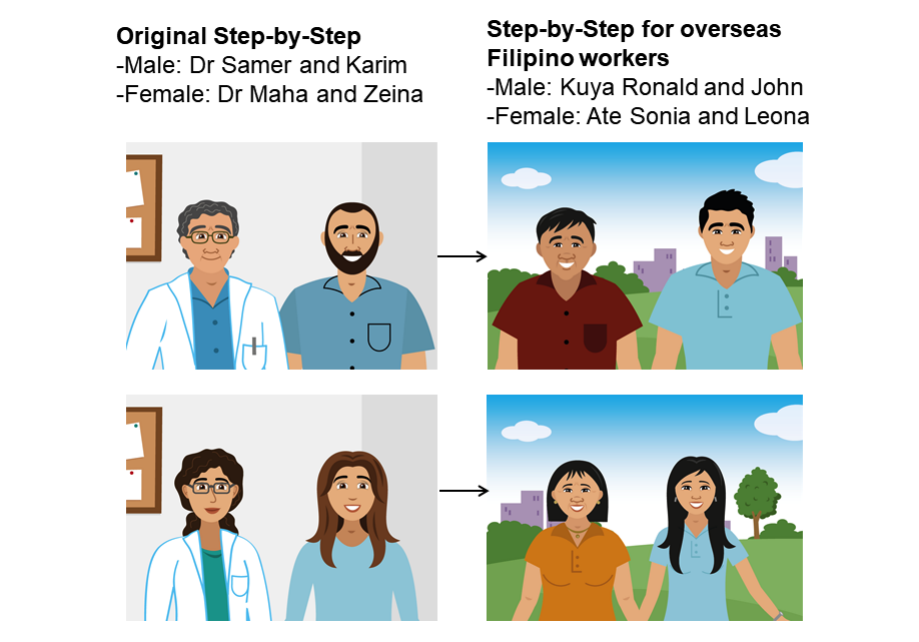
Illustration of characters in the original Step-by-Step version and in Step-by-Step for overseas Filipino workers.

To address the issue of stigma, we made 3 modifications. First, instead of stating the original program goal of “helping the user cope with difficult emotions and problems” explicitly, we changed it to “helping the user become a successful OFW, for their families’ sake.” The latter deemphasizes the focus on mental health and increases emphasis on positive goals and outcomes. The latter also still addresses the original goal but focuses on a common and integral OFW experience and value of working abroad to contribute to family’s expenses, at times as the family’s sole provider. Second, we developed the character that explains Step-by-Step concepts to the user (ie, stress and sadness) into an older OFW who has been successful in his or her work rather than a medical or mental health professional (refer to Figure 3). Changing the character to a fellow OFW normalizes the experience of problems and removes the stigma. Furthermore, interview and FGD data showed that making the character successful incorporates the aspirations of the target group. Third, we changed text such as “suffering at the moment” to “stressed at the moment” to make them sound less grave and more normative, as advised by FGD participants.

**Figure 4:**
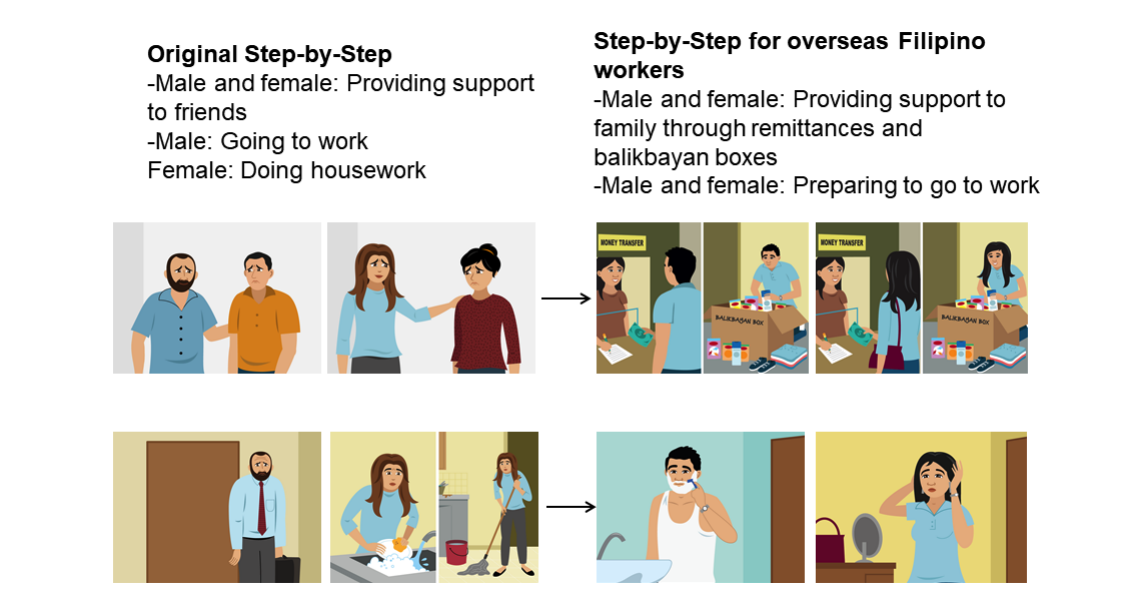
Illustration of providing support to others in the original Step-by-Step version and in Step-by-Step for overseas Filipino workers.

Some of the content was removed from Step-by-Step or changed. We removed certain hand gestures and drug use as a negative coping strategy to make the program apolitical. We removed the illustration and text about a character staying in bed all day as a negative coping strategy because this is an acceptable, even helpful, coping strategy for OFWs. We changed the illustration on characters doing household chores to sending remittances in a bank and filling up a package or what Filipinos call *balikbayan box* (literal translation: back to country) to the Philippines and preparing to go to work (refer to Figure 4) to be more neutral and suitable across occupations. These are ubiquitous OFW experiences, regardless of job or socioeconomic status.

Another is “drinking alcohol,” which we changed to “drinking too much alcohol.” Participants recommended adding the term “too much” to make it a negative coping strategy.

**Figure 5:**
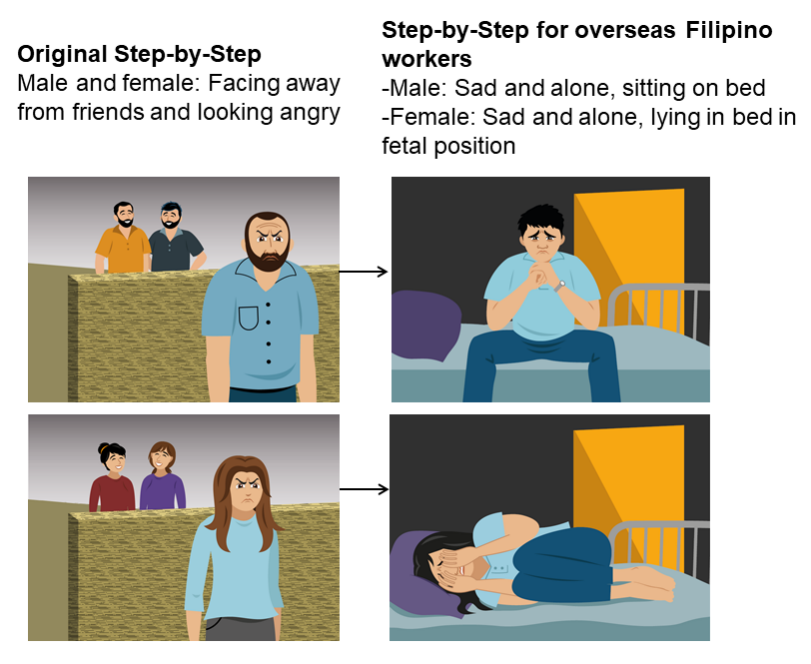
Illustration of isolating oneself from others in the original Step-by-Step version and in Step-by-Step for overseas Filipino workers.

We also made considerations for the male version. As much as possible, we ensured that female and male versions had similar storylines, activities, and illustrations so as to limit gender stereotyping as much as possible. However, for some illustrations, we made them slightly different to appeal to male OFWs by avoiding what male FGD participants believed to look too feminine. For example, in a scene that shows the character is isolated from others, the male character is in his bedroom looking lonely, with legs in v-position, elbows resting on his thighs, and hands clasped together. In the female version, the character is still alone in her bedroom but is in fetal position and hands covering her face (refer to Figure 5).

### Relevance

There were 4 issues with regard to the relevance of the original text and illustrations of Step-by-Step. The first issue was in terms of the characters. The generic version used Lebanese and Muslim characters (ie, characters had names like Zeina and Karim, females wore hijab, and males had thick beards; refer to Figure 3), which were different from typical Filipino names and appearance. The second issue was in terms of the lack of relatable values in the stories. For example, participants found the tone of the stories and personality of the characters to be too serious and robotic, which they felt were not attuned to the Filipino value of showing warmth and hospitality. The third and fourth issues were in terms of the lack of relatable problems and activities. Some of the problems in the generic version included destruction of one’s home and community. Although these happen to some OFWs, participants felt these were not the most pertinent problems in their community. Moreover, participants deemed some of the activities in the generic version atypical or unfeasible, such as planting herbs and spending time with family. These were irrelevant to OFWs because while abroad, there is no ample space to plant herbs, and OFWs cannot be physically together with their families. Participants added that planting herbs would not come across as a fun activity for many OFWs.

To increase relevance, we made the following adaptations: (1) choosing appropriate names and appearance of main characters, (2) highlighting Filipino values, (3) using relatable problems, and (4) using relatable activities.

We first developed the characters’ names and appearances to make them sound and look Filipino. The older characters had Spanish-sounding names (Kuya Ronald and Ate Sonia), whereas the younger characters had more modern and American-sounding names (John and Leona), both of which are common naming practices that are related to the colonial and changing cultural context of the Philippines. We also ensured that the names had positive meanings (ie, a wise person and a fighter) and were approved or chosen by the participants. For the older characters, we added the terms *Ate* or *Kuya* before their names, which mean respected older sister or older brother in Filipino, often associated with being sensible and experienced. For the characters’ appearances (refer to Figure 3), participants wanted their hair straight and black and their skin color warm or olive in tone. The participants also wanted the characters to look successful as they link being a migrant for years to having saved money and made investments but added that they needed to dress comfortably as they are busy at work. They proposed making their clothing simple, comfortable but modern, and adorned with jewelry such as watches for the male characters and earrings, necklaces, and watches for the female characters.

We highlighted Filipino values in 3 ways. First, experts and participants advised adapting the personality of the program and characters to exude desirable Filipino values of family orientation, showing warmth and care for others, sociability, and positive thinking. Participants added changing the tone of the text to become more conversational and story-like to sound more engaging and realistic such that the characters seem to be talking to the users. Participants also recommended that the characters addressed the user as *kabayan* or countryman, a common term used among OFWs, which denotes similarity and familiarity with fellow Filipino migrants. The older characters also acted as mentors or coaches to the younger characters and user. The younger characters called the user *sis* or *bro* to denote kinship. They showed eagerness to share their stories and emotions with the users and to learn and become better. Participants advised for all characters to offer encouragement or reassurance to the user, with lines such as “Keep up the good work!” and “You can do it!” They recommended matching these with illustrations where the characters gave warm smiles to welcome and bid the users goodbye and thumbs-up sign to show approval.

Second, the texts were changed to highlight that OFWs consider their family as motivation for going abroad and working hard, even making sacrifices for them. Participants suggested matching the illustrations by showing OFWs thinking of their families often and missing them. Furthermore, participants wanted to emphasize being with friends and family as crucial to one’s mental health and as culturally appropriate and expected. Participants proposed matching the text with illustrations of characters spending time and having fun with friends who were also OFWs and with family through Web-based communication.

Third and last, as suggested by all 3 experts during individual interviews, we mentioned additional Filipino values such as *bayanihan* and *utang-na-loob* in the stories. *Bayanihan* means working together to help someone, which we added in an activity where an OFW helped a main character in a task. *Utang-na-loob* means debt of gratitude, which we mentioned as a motivator for a main character to reach out to a friend who helped them in the past.

We adapted the content in terms of the problems the characters experienced to make them more typical, based on interview and FGD data. Examples of problems included leaving their family behind, having personal conflicts, and having too much work or having no break or day off.

**Figure 6:**
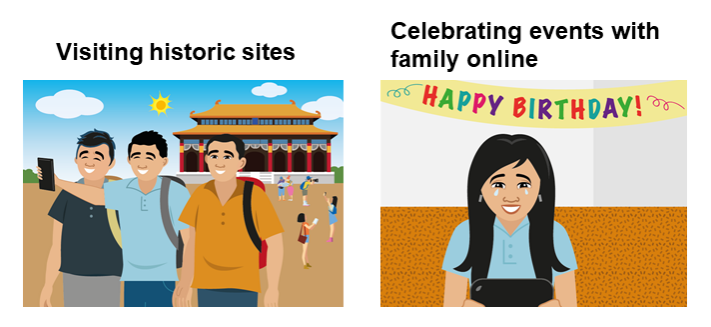
Illustration of enjoyable activities in Step-by-Step for overseas Filipino workers.

We likewise adapted the content in terms of the activities the characters engaged in to make them enjoyable and doable to as many OFWs as possible. On the basis of FGD data, examples include eating *merienda* or afternoon snacks with friends, visiting nearby historic sites, celebrating events with family using Web-based communication, and singing videoke (video karaoke) with friends (refer to Figure 6). These are typical fun activities that Filipinos engage in. Filipinos are fond of eating meals together, exploring new sites when abroad, connecting with families back home, and listening to music and singing. These activities are also relatively inexpensive and not that time-consuming, which make them feasible to do during days off and even with limited finances.

### Comprehensibility

The generic version of Step-by-Step had elements that were incomprehensible, unclear, or too complex. In the text, there were sentences that participants deemed too long or repetitive and, therefore, difficult to understand. Some participants expressed finding some terms (ie, *peers*, *pace*, and *social support*) complicated. Finally, participants found that some texts and illustrations were not clearly linked. For example, one passage in the text was about isolating oneself from other people when one feels sad. Although participants understood the text, they mistook the illustration where the character is facing away from 2 other characters as friends gossiping about the character (refer to Figure 5).

To make the adaptation comprehensible, we used Filipino and English languages. Filipino is the national language in the Philippines. Both Filipino and English are official languages and are widely spoken by OFWs as primary mediums of communication, that is, Filipino with fellow OFWs who come from different regions in the Philippines and English with their employers. Filipino is derived from Tagalog, the main language of 35.1% of households in the Philippines [30].

On the basis of FGD data, we also simplified the text in 3 ways to boost comprehension: shortening long sentences by removing words, dividing long sentences into 2 sentences, and using simplest words and phrases (ie, changed the word *peers* to *friends* and *pace* to *speed*). When a term or an idea was too abstract, we changed the words or added extra words or lines to clarify what they meant. For example, instead of the abstract concept of *social support*, we used *helping hands* as suggested during an FGD because although participants understood what social support meant, they could not verbalize their understanding of the term.

For the Filipino version, participants suggested simplifying text by removing sentences when paragraphs sounded repetitive and confusing. We retained those lines in the English version as they did not sound repetitive in English. Moreover, some English words or phrases when translated to Filipino sounded awkward; hence, we retained the original words in English. For example, we retained the word *congratulations* because its Filipino translation of *binabati kita* or “I compliment you” sounded odd to participants. We removed English words or phrases that did not translate well into Filipino, as suggested by participants.

We adapted texts and illustrations that were not clearly linked, following participants’ recommendations. For example, for the illustration about isolating oneself, we had the illustration changed so that the character is in bed alone (refer to Figure 5).

### Completeness

All key concepts in the original version are intact in the adapted Step-by-Step for OFW. We also made sure that the English and Filipino versions were as similar as possible, so that if an OFW chooses to use the English version, they would have an equivalent program to the Filipino version and vice versa. For the Filipino version, instead of translating all English words to Filipino, we retained some English words because OFWs use these words to describe their experiences. The combination of Filipino and English (ie, Taglish) is an unofficial language [30] that is normative and accepted among Filipinos. An example is being *homesick* as they are away from their family. Participants recommended retaining the English word *homesick* instead of using the Filipino translation of *hinahanap-hanap ang pamilya* (yearning for family) because the word homesick is a common OFW term and is deeply tied to working abroad far from one’s family.

## Discussion

### Principal Findings

To our knowledge, this is the first study that culturally adapted an e-mental health intervention for the OFW population and Filipinos generally. We illustrated how the 4-point framework to improve acceptance, relevance, comprehensibility, and completeness is useful in navigating the process of culturally adapting the Step-by-Step program. It was a practical guide to both us, the researchers, and the participants as it enabled us to capture and flesh out crucial elements to make the finished product attuned and sensitive to the context and experiences of OFWs. In turn, we were able to make the program culturally specific for this group, which is essential among the 4 features of effective e-mental health interventions [15].

### Lessons Learned

For future cultural adaptations, we recommend that all illustrations and content be adapted at the same time. In our experience, participants were more engaged in the presence of visual stimuli (recall that we were not able to present illustrations for sessions 0-2). Furthermore, it was easier for them to understand the texts and key concepts while being able to see the illustrations.

We recommend that the same set of participants be part of the FGDs from start to finish to avoid spending time explaining the program, the mechanics of the cognitive interviews and discussion, and details from previous sessions. We were able to do this with the majority of our FGDs, but scheduling limitations hindered participants from attending all sessions. Therefore, to accommodate the realities of the population, we allowed the FGDs to be open to newcomers. Some participants were more able to provide input than others. As in any FGD, it is important to select participants who are more open, less shy, and comfortable to freely share their thoughts. In the context of this adaptation study, norms of social hierarchy and harmony within the cultural group may have influenced how much people shared within the groups.

Another limitation is the lack of standard and systematic evaluation on whether the inputs from members of the FGD represent the entire community. Further investigation is also recommended to determine which elements were most salient to the participants to more clearly know when adaptations are good enough for the cultural group.

### Relevance of the Research

The methods and detailed results of formative adaptation work as described in this study are often not shared by researchers or program implementers in mainstream literature [9,11]. As it is such a crucial step in planning and care provision, we hope that this study will not only highlight the importance of cultural adaptation but also provide the audience a replicable account of how to conduct such formative research.

Following the Medical Research Council Guidelines for Complex Interventions and the WHO scalable psychological interventions program [31,32], this adapted program will be rigorously evaluated. The initial feasibility trial and subsequent full-scale randomized controlled trial both include process evaluation components. Subsequent changes to the illustrations, content, and program story will be made, if needed.

## Abbreviations

EBT: evidence-based treatment
e-mental: electronic mental
FGD: focus group discussion
NGO: nongovernmental organization
OFW: overseas Filipino worker
SAR: Special Administrative Region
